# The accurate diagnosis for COVID-19 variants using nearly initial-rough sets

**DOI:** 10.1016/j.heliyon.2024.e31288

**Published:** 2024-05-17

**Authors:** Radwan Abu-Gdairi, Mostafa K. El-Bably

**Affiliations:** a Department of Mathematics, Faculty of Science, Zarqa University, Zarqa 13110, Jordan; b Department of Mathematics, Faculty of Science, Tanta University, Tanta 31527, Egypt

**Keywords:** Initial-neighborhoods, Rough sets, Topology, Nearly open sets, COVID-19, 54A10, 68W25, 68U01, 68U35, 92C50

## Abstract

The rapid evolution of rough-set theory has prompted the need for enhanced methodologies in medical diagnostics, particularly regarding COVID-19 variant detection. This study introduces refined mathematical techniques based on topological structures (called nearly initial-rough sets) derived directly from initial-rough sets. Four categories of rough-set methodologies are presented, demonstrating heightened accuracy through comprehensive comparisons against existing methods. By leveraging these techniques, a rule-based classification system for COVID-19 variants is established, achieving 100 % accuracy measures through rigorous testing against real-world and computer-generated data. The implications of these advancements in medical diagnosis hold promise for future research, offering accessible and precise tools for variant identification and prediction. Using a medical application as a case study, we demonstrate superiority through comparative analyses, aligning mathematical results with medical data and showcasing the potential for broader applications beyond experts in topology. Furthermore, the study outlines an algorithm simplifying implementation, particularly in MATLAB, and suggests future explorations in medical, economic, and diverse theoretical frameworks to enhance applicability.

## 1 Introduction

In recent years, the growing attention towards rough set theory and its extended models, particularly in computer science and artificial intelligence, has been notable. Initially introduced by Pawlak [1,2] in 1982, this theory has proven to be an effective tool for handling problems with imperfect knowledge. It functions by classifying objects based on an equivalence relation, capturing the completeness of information within a set. The fundamental principles of this theory involve approximation operators and accuracy measures, providing decision-makers with essential data regarding the structure and size of boundary regions. However, the strict requirement of an equivalence relation imposes limitations on the conventional applications of rough set theory.

To overcome these limitations, various generalizations of the theory have emerged, utilizing either arbitrary or specific relations. Yao [3] pioneered this line of research in 1998, where researchers assumed specific relations, leading to various types of generalized rough sets, including tolerance [4], similarity [5,6], quasi-order [7], and a general relation [[8], [9], [10]]. In 2014, Abd El-Monsef et al. [11] introduced the concept of the j-neighborhood space (abbreviated as j**-**NS), a generalized form of the neighborhood space derived from a binary relation. This framework extended Pawlak's rough sets by utilizing various topologies induced by the j**-**NS.

Subsequently, numerous authors have explored operations involving the j**-**NS to uncover new types of neighborhood systems. For instance, Atef et al. [12] defined the j-adhesion neighborhood space, demonstrating the potential of the j**-**NS and the adhesion concept [13] as a valuable tool in exploring and advancing neighborhood-based concepts which proposed by relations in [[14], [15]]. However, discrepancies in some results presented by El-Bably et al. [16] regarding j-adhesion neighborhoods were later corrected, offering new insights into these techniques.

Moreover, El-Bably and Al-shami [17] introduced core minimal-neighborhoods using binary relations, extending Pawlak rough sets to four different types of generalized rough sets and applying them to medical applications concerning lung cancer diseases. The topologies generated by Abd El-Monsef et al.'s method [11] has paved the way for more topological applications in rough sets, such as in medicine [[18], [19], [20], [21]], and economy [22,23].

Recent studies, particularly the work of El-Bably et al. [24], have utilized the j**-**NS to investigate generalized closure spaces derived from binary relations. These studies have introduced new levels of granularity within rough sets and have been extensively referenced in practical applications. In various research works, topological structures have played a significant role in extending rough set theories, including trends like soft rough sets [[25], [26], [27]] and rough fuzzy sets [28]. Additionally, leveraging the concept of the j**-**NS, M. Hosny [29] introduced novel approaches to rough sets derived from ideal structures. Subsequent enhancements and adjustments to these techniques were proposed by R. A. Hosny et al. [30]. Furthermore, other papers have also employed topological properties to define rough sets [[31], [32], [33], [34], [35]].

As advancements in computational theory progress, the emergence of COVID-19 as a global pandemic has profoundly disrupted normal life worldwide [[35], [36], [37], [38]]. The escalating numbers of active and fatal cases have significantly impacted the psychological well-being of individuals. The development of various variants, notably the designated variants of concern (VoC) such as Alpha, Beta, Gamma, Delta, and Omicron, has been linked to rapid transmission, severe infections, and fatalities. The unpredictability of these variants, along with their potential mutations, poses a formidable challenge for healthcare systems. With the virus expected to become endemic, there is an ongoing necessity to enhance the identification, prediction, and classification of COVID-19 variants.

The recent advancement of technology has presented challenges and opportunities for researchers to automate COVID-19 variant identification and prediction [38]. The accurate diagnosis of COVID-19 variants holds paramount importance in determining appropriate treatment and preventive measures. Thus, the core objective of this paper is to introduce new tools, based on topological structures known as "nearly open concepts," which offer a refined granulation of rough sets to aid in the precise medical diagnosis of COVID-19 variants.

Building on the groundwork laid by prior research, we extend these methods to develop more accurate techniques based on near-open concepts, directly derived from the binary relation. Unlike previous approaches relying on induced topology, our proposed methods are designed for ease of application, even by individuals not specialized in topology. These methodologies open avenues for broader topological applications across various disciplines. The importance of presenting topological approximations independently of delving into the complexities of topology is pivotal for establishing rough approximations (initial-approximations) without direct reliance on intricate topological concepts. This approach facilitates a streamlined pathway for researchers, particularly those lacking an extensive background in topology, enabling them to readily apply rough-set theories in their scientific inquiries. By defining these approximations without mandating an in-depth understanding of topology, the aim is to democratize these powerful tools, rendering them more accessible and user-friendly. This empowerment broadens the horizon for researchers, offering them the opportunity to harness the potential of rough sets in their work. While methodologies grounded in topology offer considerable advantages, particularly in crafting specialized algorithms for diverse fields and real-world applications, the creation of specific algorithms that seamlessly integrate topological principles can effectively bridge gaps and resolve discrepancies in optimizing algorithms for practical utility.

Our contributions include a fundamental method to generate topological initial-rough sets without relying on induced topology, leading to the construction of different generalized rough sets directly from the binary relation. These approaches are founded on defining near-open concepts through initial approximations and their extensions. Comparative analyses demonstrate the superior accuracy of nearly initial-approximations over other existing methodologies. Finally, we employ these methodologies to create a robust rule base for classifying and predicting COVID-19 variants, providing efficient decision-making tools for medical practitioners and saving crucial time and resources.

By applying these proposed techniques to real-world data systems, we validate their effectiveness in accurately identifying COVID-19 variants and aligning mathematical results with medical observations. Our method represents a mathematical approach to enhancing accuracy in decision-making and revealing hidden patterns within data, thus contributing to the ongoing efforts to combat the challenges posed by evolving COVID-19 variants.

The manuscript presents our primary contributions through several key stages.

1. **Introduction of Methodologies:** We introduce pivotal methodologies for generating topological initial-rough sets, eliminating dependence on induced topology. This enables the direct formation of diverse generalized rough sets from binary relations. Our novel concept of "nearly initial-approximations" showcases superior accuracy compared with established methods. Extensive comparisons and counter-examples against prior approaches substantiate this assertion.
2. **Rule-Based Classification System for COVID-19 Variants:** Leveraging these methodologies, we establish a fundamental rule-based classification system crucial for the precise identification and prediction of COVID-19 variants. Through the application of these techniques to real-world data [38], we demonstrate the efficacy of "nearly initial-approximations" in facilitating accurate decision-making. This alignment of mathematical results with medical diagnoses reveals latent patterns within the dataset.
3. **Significance:** Our work constitutes a significant mathematical breakthrough. It not only amplifies decision-making accuracy but also provides a comprehensive methodology for decoding COVID-19 variants. This approach holds promise in conserving invaluable time and resources for patients and clinicians alike.
4. **Comparative Analysis with Existing Methods:** Investigating and analyzing these newly introduced nearly initial-approximations by comparing them with other established methods present in the literature. This comparison could highlight the advantages or distinct features of the proposed techniques.
5. **Algorithm and Framework Proposal:** Additionally, the manuscript proposes an algorithm and framework tailored for the implementation of these methods in decision-making problems. It delineates an algorithm that simplifies implementation, specifically in MATLAB. Moreover, it suggests future explorations across medical, economic, and diverse theoretical frameworks to augment the applicability of these methodologies. This expansion aims to handle future big data challenges and broaden the scope of applications.

## 2 Basic concepts

In this section, we outline the main ideas about some previous studies of rough sets (namely, Yao [3], Abd El-Monsef et al. [11], Dai et al. [6], El-Sayed et al.[19], and Abu-Gadairi [39]).

### 2.1 Yao approaches (1998)

Definition 2.1 [3] Let R be a binary relation on a non-empty finite set U. The Yao-lower and Yao-upper approximations of M are defined respectively by:

$$\underline{R}\left(M\right)=\left\{m\in U|mR\subseteq M\right\},\text{and}\;\bar{R}\left(M\right)=\left\{m\in U|mR\cap M\neq \varphi \right\}\text{.}$$

Where mR represents the after set (right neighborhood) of m.

Moreover, the Yao-boundary region, and Yao-accuracy measure are given respectively by:

$B\left(M\right)=\bar{R}\left(M\right)-\underline{R}\left(M\right)$, and $\mu \left(M\right)=\frac{\left|\underline{R}\left(M\right)\right|}{\left|\bar{R}\left(M\right)\right|}$, where $\left|\bar{R}\left(M\right)\right|\neq 0$.

Evidently, 0≤μ(M)≤1, and if μ(M)=1, then M is Yao-exact. Otherwise, it is Yao-rough.

### 2.2 Abd El-Monsef et al. Approaches (2014)

Abd El-Monsef et al.'s approaches [11] focus on summarizing the fundamental concepts of j-**NS** and j-approximations for specific cases of j. Additionally, we establish new results and elucidate relationships between the approaches outlined by Abd El-Monsef et al. and Yao approximations [3].

Definition 2.2 [11] Let R be a binary relation on a non-empty finite set U. The j-neighborhood of $x\in U,\text{denoted}\;\text{by}\;n_{j}\left(x\right),j\in \left\{r,l,⋏,⋎\right\}$, can be defined as follows:

(i)r-neighborhood [3]: $n_{r}\left(x\right)=\left\{y\in U\;|\;xRy\right\}$.
(ii)l-neighborhood $:n_{l}\left(x\right)=\left\{y\in U\;|\;yRx\right\}$.
(iii)⋏-neighborhood: $n_{⋏}\left(x\right)=n_{r}\left(x\right)\cap n_{l}\left(x\right)$.
(iv)⋎-neighborhood: $n_{⋎}\left(x\right)=n_{r}\left(x\right)\cup n_{l}\left(x\right)$.

Definition 2.3 [11] Let R be a binary relation on U and $\xi_{j}:U⟶℘\left(U\right)$ be a mapping that assigns, for each x in U, its j-neighborhood in ℘(U), where ℘(U) denotes the power set of U. The triple $\left(U,R,\xi_{j}\right)$ is termed a j-neighborhood space (abbreviated as j**-**NS).

Theorem 2.1 [11] If $\left(U,R,\xi_{j}\right)$ is a j**-**NS, then for each j∈{r,l,⋏,⋎}, the collection

$T_{j}=\left\{M\subseteq U|\;\forall \;m\in M,n_{j}\left(m\right)\subseteq M\;\right\}$ forms a topology on U.

Definition 2.4 [11] Consider $\left(U,R,\xi_{j}\right)$ to be a j**-**NS. A subset M⊆U is termed j**-**open if $M\in T_{j}$, and the complement of an j**-**open set is j**-**closed. The family $F_{j}$ encompassing all j-closed sets in a j**-**NS is defined as $F_{j}=\left\{\;F\subseteq U\;|\;F^{c}\in T_{j}\right\}$.

Definition 2.5 [11] Considering $\left(U,R,\xi_{j}\right)$ as a j**-**NS and M⊆U. The j-lower and j-upper approximations of M are given respectively as ${\underline{R}}_{j}\left(M\right)=\cup \left\{G\in T_{j}:G\subseteq M\right\}={\mathrm{int}}_{j}\left(M\right)$ and ${\bar{R}}_{j}\left(M\right)=\cap \left\{H\in F_{j}:M\subseteq H\right\}={\mathrm{cl}}_{j}\left(M\right)$, where ${\mathrm{int}}_{j}\left(M\right)$ (resp. ${\mathrm{cl}}_{j}\left(M\right)$) represents the j-interior (resp. j-closure) of M.

Additionally, the j-boundary, j-positive and j-negative regions of M are given respectively by

$$B_{j}\left(M\right)={\bar{R}}_{j}\left(M\right)-{\underline{R}}_{j}\left(M\right),{POS}_{j}\left(M\right)={\underline{R}}_{j}\left(M\right),\text{and}\;{NEG}_{j}\left(M\right)=U-{\bar{R}}_{j}\left(M\right)\text{.}$$

The j-accuracy of the approximations of M⊆U is defined by

$$\mu_{j}\left(M\right)=\frac{\left|{\underline{R}}_{j}\left(M\right)\right|}{\left|{\bar{R}}_{j}\left(M\right)\right|},\text{where}\;\left|{\bar{R}}_{j}\left(M\right)\right|\neq 0\text{.}$$

Clearly, $0\leq \mu_{j}\left(M\right)\leq 1$, and if $\mu_{j}\left(M\right)=1$, then M is a j-exact set. Else, it is j-rough.

Remark 2.1 It should be noted that j-approximations (in the case of j=r) differ significantly from Yao approximations in general. For any set M:

$${\underline{R}}_{r}\left(M\right)\neq \left\{m\in U|n_{r}\left(m\right)\subseteq M\right\}=\underline{R}\left(M\right),\text{and}$$

$${\bar{R}}_{r}\left(M\right)\neq \left\{m\in U|n_{r}\left(m\right)\cap M\neq \varphi \right\}=\bar{R}\left(M\right)\text{.}$$

Examples 2.1 & 2.2 illustrate this distinction.

The following results elucidate the conditions under which Yao approaches equal Abd El-Monsef et al.'s methods.

Lemma 2.1 Let R be a transitive relation on U, and v,w∈U. If $v\in n_{r}\left(w\right)$, then $n_{r}\left(v\right)\subseteq n_{r}\left(w\right)$.

**Proof:** First, if $v\in n_{r}\left(w\right)$, then wRv (1)

Now, let $m\in n_{r}\left(v\right)$. Then vRm (2)

By the transitivity of R, using (1) and (2), we obtain wRm, which implies $m\in n_{r}\left(w\right)$. Accordingly, $n_{r}\left(v\right)\subseteq n_{r}\left(w\right)$. ∎:

Theorem 2.2 Consider $\left(U,R,\xi_{j}\right)$ as a j**-**NS and M⊆U. If R is a reflexive and transitive relation, then, the following statements hold:

(i) $\underline{R}\left(M\right)=\left\{m\in U|n_{r}\left(m\right)\subseteq M\right\}=\cup \left\{G\in T_{j}|G\subseteq M\right\}={\underline{R}}_{r}\left(M\right)$.
(ii) $\bar{R}\left(M\right)=\left\{m\in U|n_{r}\left(m\right)\cap M\neq \varphi \right\}=\cap \left\{H\in F_{j}|M\subseteq H\right\}={\bar{R}}_{r}\left(M\right)$.

**Proof:** The first item will be proven, and the others will follow similarly.

#### 2.2.1 Necessity condition

(3) $$\text{Let}\;m\in \underline{R}\left(M\right),\text{then}\;n_{r}\left(m\right)\subseteq M$$

(4) $$\text{By}\;\text{reflexivity}\;\text{of}\;R,\text{we}\;\text{get}\;m\in n_{r}\left(m\right)$$

(5) $$\text{By}\;\text{transitivity}\;\text{of}\;R,\text{we}\;\text{get}\;\forall z\in n_{r}\left(m\right),n_{r}\left(z\right)\subseteq n_{r}\left(m\right)$$

From (1), (2), and (3), we obtain $n_{r}\left(m\right)$ is an r-open set contained in M such that $m\in n_{r}\left(m\right)$, implying $m\in {\underline{R}}_{r}\left(M\right)$.

#### 2.2.2 Sufficiency condition

Let $m\in {\underline{R}}_{r}\left(M\right)$, then $\exists G\in T_{r}$ such that G⊆M and m∈G. Therefore, ∀z∈G, $n_{r}\left(z\right)\subseteq G$ which implies $n_{r}\left(m\right)\subseteq M$. Hence, $m\in \underline{R}\left(M\right)$. ∎:

The following examples illustrate that the conditions of reflexivity and transitivity of a relation are necessary and cannot be ignored.

Example 2.1 Consider U={t,u,v,w} and the reflexive relation R={(t,t),(t,u), (u,u),(u,v),(v,v),(w,w)}. Consequently, $n_{r}\left(t\right)=\left\{t,u\right\}$, $n_{r}\left(u\right)=\left\{u,v\right\}$, $n_{r}\left(v\right)=\left\{v\right\}$, and $n_{r}\left(w\right)=\left\{w\right\}$. Hence, $T_{r}=\{U,\varphi ,\left\{v\right\},\left\{w\right\},\left\{u,v\right\},\left\{v,w\right\}\text{,}$ {t,u,v},{u,v,w}}, and $F_{r}=\{U,\varphi ,\left\{t\right\},\left\{w\right\},\left\{t,u\right\},\left\{t,w\right\}\text{,}$ {t,u,v},{t,u,w}}. Now, if M={t,u} and N={v,w}, then ${\underline{R}}_{r}\left(M\right)=\varphi$ and ${\bar{R}}_{r}\left(N\right)=U$. However, $\underline{R}\left(M\right)=\left\{t\right\}$ and $\bar{R}\left(N\right)=\left\{u,v,w\right\}$.

Example 2.2 Consider U={t,u,v,w} and the transitive relation R={(t,t),(t,u), (t,v),(u,v),(v,v). Consequently, $n_{r}\left(t\right)=\left\{t,u,v\right\}$, $n_{r}\left(u\right)=\left\{v\right\}$, $n_{r}\left(v\right)=\left\{v\right\}$, and $n_{r}\left(w\right)=\varphi$. Hence, $T_{r}=\{U,\varphi ,\left\{v\right\},\left\{w\right\},\left\{u,v\right\},\left\{v,w\right\}\text{,}$ {t,u,v},{u,v,w}}, and $F_{r}=\{U,\varphi ,\left\{t\right\},\left\{w\right\},\left\{t,u\right\},\left\{t,w\right\}\text{,}$ {t,u,v},{t,u,w}}. Now, if M={v,w}, then ${\underline{R}}_{r}\left(M\right)=\left\{v,w\right\}$ and ${\bar{R}}_{r}\left(M\right)=U$. However, $\underline{R}\left(M\right)=\left\{u,v,w\right\}$ and $\bar{R}\left(M\right)=\left\{t,u,v\right\}$. Additionally, $\underline{R}\left(U\right)=U$, $\bar{R}\left(U\right)=\left\{t,u,v\right\}$, $\underline{R}\left(\varphi \right)=\left\{w\right\}$, and $\bar{R}\left(\varphi \right)=\varphi$, even though ${\underline{R}}_{r}\left(M\right)={\bar{R}}_{r}\left(M\right)=U$ and ${\underline{R}}_{r}\left(\varphi \right)={\bar{R}}_{r}\left(\varphi \right)=\varphi$.

### 2.3 Dai et al. Approaches (2018)

Definition 2.6 [6] Let R be a binary relation on U. For each w∈U, the maximal-neighborhood of it is given by

$$n_{m}\left(w\right)=\left\{ \begin{array}{c} \underset{w\in n_{r}\left(y\right)}{⋃}n_{r}\left(y\right),\text{if}\;\exists y\;\text{such}\;\text{that}\;w\in n_{r}\left(y\right).\;\\ \varphi ,\text{Other}\;\text{wise}. \end{array}\right.$$

Definition 2.7 [6] Let R be a binary relation on U. The m-lower and m-upper approximations of S⊆U are given respectively by

$${\underline{R}}_{m}\left(S\right)=\left\{w\in U:n_{m}\left(w\right)\subseteq S\right\}\;\text{and}\;{\bar{R}}_{m}\left(S\right)=\left\{w\in U:n_{m}\left(w\right)\cap S\neq \varphi \right\}\text{.}$$

Moreover, the m-positive, m-negative, and m-boundary regions, and the m-accuracy of m-approximations of S⊆U are defined respectively by

$${POS}_{m}\left(S\right)={\underline{R}}_{m}\left(S\right),{NEG}_{m}\left(S\right)=U-{\bar{R}}_{m}\left(S\right),B_{m}\left(S\right)={\bar{R}}_{m}\left(S\right)-{\underline{R}}_{m}\left(S\right),\text{and}$$

$\mu_{m}\left(S\right)=\frac{\left|{\underline{R}}_{m}\left(S\right)\right|}{\left|{\bar{R}}_{m}\left(S\right)\right|}$, where $\left|{\bar{R}}_{m}\left(S\right)\right|\neq 0$.

Additionally, if $\mu_{m}\left(S\right)=1$ then S is m-exact. If $\mu_{m}\left(S\right)\neq 1$, S is m-rough.

### 2.4 Abu-Gdairi approaches (2023)

This section offers a summary of the Abu-Gdairi methods, pivotal extensions building upon the techniques introduced by El-Syaed et al. [19]. It is dedicated to extending the concept of the 'initial-neighborhood [19]' to generalize and create four distinct topologies derived from neighborhoods.

Definition 2.8 [39] Let R be a binary relation on U. Then, we define the following neighborhoods of x∈U:

(i) r-initial neighborhood [19]: $n_{r}^{i}\left(x\right)=\left\{y\in U\;|\;n_{r}\left(x\right)\subseteq n_{r}\left(y\right)\right\}$.
(ii) l-initial neighborhood: $n_{l}^{i}\left(x\right)=\left\{y\in U\;|\;n_{l}\left(x\right)\subseteq n_{l}\left(y\right)\right\}$.
(iii) ⋏-initial neighborhood: $n_{⋏}^{i}\left(x\right)=n_{r}^{i}\left(x\right)\cap n_{l}^{i}\left(x\right)$.
(iv) ⋎-initial neighborhood: $n_{⋎}^{i}\left(x\right)=n_{r}^{i}\left(x\right)\cup n_{l}^{i}\left(x\right)$.

The following lemmas present the main properties of the above neighborhoods.

Lemma 2.2 [39] Let R be a binary relation on U. Then:

(i) ${x\in n}_{j}^{i}\left(x\right)$, for each j∈{r,l,⋏,⋎}.
(ii) If $y\in n_{j}^{i}\left(x\right)$, then $n_{j}^{i}\left(y\right)\subseteq n_{j}^{i}\left(x\right)$, for each j∈{r,l,⋏}.

Lemma 2.3 [39] Let R be a binary relation on U. Then, ∀x∈U:

(i) $n_{⋏}^{i}\left(x\right)\subseteq n_{r}^{i}\left(x\right)\subseteq n_{⋎}^{i}\left(x\right)$.
(ii) $n_{⋏}^{i}\left(x\right)\subseteq n_{l}^{i}\left(x\right)\subseteq n_{⋎}^{i}\left(x\right)$.

Next lemma illustrates the relationships between the initial-neighborhoods and j-neighborhoods.

Lemma 2.4 [39] Let $\left(U,R,\xi_{j}\right)$ be a j**-**NS and R be a reflexive and symmetric relation. Then, for each j∈{r,l,⋏,⋎}, $n_{j}^{i}\left(x\right)\subseteq n_{j}\left(x\right)$, ∀x∈U.

The following result (depends on Theorem 2.1.) discusses an interesting method to generate different topologies using the initial-neighborhoods.

Theorem 2.3 [39] If $\left(U,R,\xi_{j}\right)$ is a j**-**NS. Then, for each j∈{r,l,⋏,⋎}, the collection:

$T_{j}^{i}=\left\{M\subseteq U|\;\forall \;m\in M,n_{j}^{i}\left(m\right)\subseteq M\;\right\}$ forms a topology on U.

The next proposition gives the relationships among different topologies $T_{j}^{i}$.

Proposition 2.1 [39] If $\left(U,R,\xi_{j}\right)$ is a j**-**NS. Then:

(i) $T_{⋎}^{i}\subseteq T_{r}^{i}\subseteq T_{⋏}^{i}$. **(ii)**$T_{⋎}^{i}\subseteq T_{l}^{i}\subseteq T_{⋏}^{i}$.

Proposition 2.2 [39] If $\left(U,R,\xi_{j}\right)$ is a j**-**NS and R be a reflexive and symmetric relation. Then, for each j∈{r,l,⋏,⋎}: $T_{j}\subseteq T_{j}^{i}$

Definition 2.9 [39] Let $\left(U,R,\xi_{j}\right)$ be a j**-**NS. A subset S⊆U is termed an j-initial open set if $S\in T_{j}^{i}$. The complement of an j-initial open set is referred to as an j-initial closed set. The family $F_{j}^{i}$ encompasses all j-initial closed sets and is defined as: $F_{j}^{i}=\left\{\;F\subseteq U\;|\;F^{c}\in T_{j}^{i}\;\right\}$. Additionally, the following definitions are established:

(i) The j-initial interior of S⊆U is defined by ${\mathrm{int}}_{j}^{i}\left(S\right)=\cup \{G\in T_{j}^{i}:G\subseteq S\}$.
(ii) The j-initial closure of S⊆U is defined by ${\mathrm{cl}}_{j}^{i}\left(S\right)=\cap \{H\in F_{j}^{i}:S\subseteq H\}$.

Definition 2.10 [39] In a j**-**NS $\left(U,R,\xi_{j}\right)$ where S⊆U, the j-initial lower and j-initial upper approximations of S are respectively defined as:

$${\underline{R}}_{j}^{i}\left(S\right)={\mathrm{int}}_{j}^{i}\left(S\right)\;\text{and}\;{\bar{R}}_{j}^{i}\left(S\right)={\mathrm{cl}}_{j}^{i}\left(S\right)\text{.}$$

Additionally, the j-initial boundary, j-initial positive, and j-initial negative regions of S are given by:

$B_{j}^{i}\left(S\right)={\bar{R}}_{j}^{i}\left(S\right)-{\underline{R}}_{j}^{i}\left(S\right)$, ${POS}_{j}^{i}\left(S\right)={\underline{R}}_{j}^{i}\left(S\right)$ and ${NEG}_{j}^{i}\left(S\right)=U-{\bar{R}}_{j}^{i}\left(S\right)$, respectively.

The j-initial accuracy of the j-initial approximations of S⊆U is defined by

$$\mu_{j}^{i}\left(S\right)=\frac{\left|{\underline{R}}_{j}^{i}\left(S\right)\right|}{\left|{\bar{R}}_{j}^{i}\left(S\right)\right|},\text{where}\;\left|{\bar{R}}_{j}^{i}\left(S\right)\right|\neq 0\text{.}$$

It is evident that $0\leq \mu_{j}^{i}\left(S\right)\leq 1$, and if $\alpha_{j}^{i}\left(S\right)=1$, then S is termed an j-initial definable (j-initial exact) set; otherwise, it is termed j-initial rough.

**Note:** When j=r, the j-initial approximations correspond to the approach by El-Sayed et al. [19].

The following proposition provides a summary of the principal properties of j-initial approximations.

Proposition 2.3 [39] In a j**-**NS $\left(U,R,\xi_{j}\right)$ where S,T⊆U, the following properties hold for j-initial approximations:

$\left(1\right)\;{\underline{R}}_{j}^{i}\left(S\right)\subseteq S\subseteq {\bar{R}}_{j}^{i}\left(S\right)$.

$\left(2\right)\;{\underline{R}}_{j}^{i}\left(U\right)={\bar{R}}_{j}^{i}\left(U\right)=U$, ${\underline{R}}_{j}^{i}\left(\varphi \right)={\bar{R}}_{j}^{i}\left(\varphi \right)=\varphi$.

$\left(3\right){\bar{R}}_{j}^{i}\left(S\cup T\right)={\bar{R}}_{j}^{i}\left(S\right)\cup {\bar{R}}_{j}^{i}\left(T\right)$.

$\left(4\right){\underline{R}}_{j}^{i}\left(S\cap T\right)={\underline{R}}_{j}^{i}\left(S\right)\cap {\underline{R}}_{j}^{i}\left(T\right)$.

$\left(5\right)\;\text{If}\;S\subseteq T\;\text{then}\;{\underline{R}}_{j}^{i}\left(S\right)\subseteq {\underline{R}}_{j}^{i}\left(T\right)$.

$\left(6\right)\;\text{If}\;S\subseteq T\;\text{then}\;{\bar{R}}_{j}^{i}\left(S\right)\subseteq {\bar{R}}_{j}^{i}\left(T\right)$.

$\left(7\right)\;{\underline{R}}_{j}^{i}\left(S\cup T\right)⊇{\underline{R}}_{j}^{i}\left(S\right)\cup {\underline{R}}_{j}^{i}\left(T\right)$.

$\left(8\right){\bar{R}}_{j}^{i}\left(S\cap T\right)\subseteq {\bar{R}}_{j}^{i}\left(S\right)\cap {\bar{R}}_{j}^{i}\left(T\right)$.

$\left(9\right)\;{\underline{R}}_{j}^{i}\left(S\right)={\left[{\bar{R}}_{j}^{i}\left(S^{c}\right)\right]}^{c}$, $S^{c}$ represents a complement of S.

$\left(10\right)\;{\bar{R}}_{j}^{i}\left(S\right)={\left[{\underline{R}}_{j}^{i}\left(S^{c}\right)\right]}^{c}$.

$\left(11\right)\;{\underline{R}}_{j}^{i}\left({\underline{R}}_{j}^{i}\left(S\right)\right)={\underline{R}}_{j}^{i}\left(S\right)$.

$\left(12\right)\;{\bar{R}}_{j}^{i}\left({\bar{R}}_{j}^{i}\left(S\right)\right)={\bar{R}}_{j}^{i}\left(S\right)$.

For more details, see [39].

## 3 Nearly initial-approximations

Within this section, new extensions of j-initial rough sets are introduced, offering alternative approximations. These constructions entail topological structures rooted in near concepts, bypassing reliance on formal Topology. Consequently, these methods potentially offer ease of comprehension for individuals not specializing in Topology. Such techniques extend the application scope of Topology across various scientific disciplines. The properties and connections among these methodologies are scrutinized through established results and illustrative examples. To commence, we reframe the definition of initial-approximations (Definition 2.10) without direct reliance on Topology, as demonstrated by the following theorem.

Theorem 3.1 Given $\left(U,R,\xi_{j}\right)$ as a j**-**NS and S⊆U, for each j∈{r,l,⋏,⋎}:

${\underline{R}}_{j}^{i}\left(S\right)=\left\{x\in U:n_{j}^{i}\left(x\right)\subseteq S\right\}$ and ${\bar{R}}_{j}^{i}\left(S\right)=\left\{x\in U:n_{j}^{i}\left(x\right)\cap S\neq \varphi \right\}$.

**Proof:** We demonstrate the first statement; the others follow similarly.

**Necessity condition:** Let x∈U such that $x\in n_{j}^{i}\left(x\right)\subseteq S$. By utilizing Lemma 2.1, $\forall y\in n_{j}^{i}\left(x\right)$, $n_{j}^{i}\left(y\right)\subseteq n_{j}^{i}\left(x\right)$, implying $n_{j}^{i}\left(x\right)\in T_{j}$, thus $x\in n_{j}^{i}\left(x\right)\subseteq S$. Consequently, $x\in {\underline{R}}_{j}^{i}\left(S\right)$.

**Sufficiency condition:** If $x\in {\underline{R}}_{j}^{i}\left(S\right)$, then there exists $D\in T_{j}$ such that x∈D⊆S. Hence, for all y∈D, $n_{j}^{i}\left(y\right)\subseteq D$ , implying x∈S and $x\in n_{j}^{i}\left(x\right)\subseteq S$. ∎:

**Note:**Theorem 3.1 presents an efficient method for computing initial-approximations directly using initial-neighborhoods, obviating the need for topology calculations.

Definition 3.1 Let $\left(U,R,\xi_{j}\right)$ be a j**-**NS and S⊆U. Then

(i) Pre-initial approximations

${\underline{P}}_{j}^{i}\left(S\right)=S\cap {\underline{R}}_{j}^{i}\left({\bar{R}}_{j}^{i}\left(S\right)\right)$ and ${\bar{P}}_{j}^{i}\left(S\right)=S\cup {\bar{R}}_{j}^{i}\left({\underline{R}}_{j}^{i}\left(S\right)\right)$.

(ii) Semi-initial approximations

${\underline{S}}_{j}^{i}\left(S\right)=S\cap {\bar{R}}_{j}^{i}\left({\underline{R}}_{j}^{i}\left(S\right)\right)$ and ${\bar{S}}_{j}^{i}\left(S\right)=S\cup {\underline{R}}_{j}^{i}\left({\bar{R}}_{j}^{i}\left(S\right)\right)$.

(iii) γ-initial approximations

${\underline{\gamma }}_{j}^{i}\left(S\right)={\underline{P}}_{j}^{i}\left(S\right)\cup {\underline{S}}_{j}^{i}\left(S\right)$ and ${\bar{\gamma }}_{j}^{i}\left(S\right)={\bar{P}}_{j}^{i}\left(S\right)\cap {\bar{S}}_{j}^{i}\left(S\right)$.

(iv) α-initial approximations

${\underline{\alpha }}_{j}^{i}\left(S\right)=S\cap {\underline{R}}_{j}^{i}\left({\bar{R}}_{j}^{i}\left({\underline{R}}_{j}^{i}\left(S\right)\right)\right)$ and ${\bar{\alpha }}_{j}^{i}\left(S\right)=S\cup {\bar{R}}_{j}^{i}\left({\underline{R}}_{j}^{i}\left({\bar{R}}_{j}^{i}\left(S\right)\right)\right)$.

(v) β-initial approximations

${\underline{\beta }}_{j}^{i}\left(S\right)=S\cap {\bar{R}}_{j}^{i}\left({\underline{R}}_{j}^{i}\left({\bar{R}}_{j}^{i}\left(S\right)\right)\right)$ and ${\bar{\beta }}_{j}^{i}\left(S\right)=S\cup {\underline{R}}_{j}^{i}\left({\bar{R}}_{j}^{i}\left({\underline{R}}_{j}^{i}\left(S\right)\right)\right)$.

Definition 3.2 Let $\left(U,R,\xi_{j}\right)$ be a j**-**NS and let S⊆U. For each N∈{P,S,γ,α,β}:

$B_{N_{j}}^{i}\left(S\right)={\bar{N}}_{j}^{i}\left(S\right)-{\underline{N}}_{j}^{i}\left(S\right)$, ${POS}_{N_{j}}^{i}\left(S\right)={\underline{N}}_{j}^{i}\left(S\right)$, ${NEG}_{N_{j}}^{i}\left(S\right)=U-{\bar{N}}_{j}^{i}\left(S\right)$, and

$\mu_{N_{j}}^{i}\left(S\right)=\frac{\left|{\underline{N}}_{j}^{i}\left(S\right)\right|}{\left|{\bar{N}}_{j}^{i}\left(S\right)\right|}$ , such that ${\bar{N}}_{j}^{i}\left(S\right)\neq \varphi$.

The above operators are called nearly initial-boundary, nearly initial-positive, and nearly initial-negative regions, and represent the nearly initial-accuracies of the approximations of S, respectively. It is clear that $0\leq \mu_{N_{j}}^{i}\left(S\right)\leq 1$. If $\mu_{N_{j}}^{i}\left(S\right)=1$, then S is called a nearly initial-definable (or nearly initial-exact) set. Otherwise, it is called a nearly initial-rough set.

Some properties of the nearly j-initial approximations are imposed in the next proposition.

Proposition 3.1 Let $\left(U,R,\xi_{j}\right)$ be a j**-**NS and let S,T⊆U. For any N∈{P,S,γ,α,β}:

$\left(1\right)\;{\underline{N}}_{j}^{i}\left(S\right)\subseteq S\subseteq {\bar{N}}_{j}^{i}\left(S\right)$.

$\left(2\right)\;{\underline{N}}_{j}^{i}\left(U\right)={\bar{N}}_{j}^{i}\left(U\right)=U$, ${\underline{N}}_{j}^{i}\left(\varphi \right)={\bar{N}}_{j}^{i}\left(\varphi \right)=\varphi$.

$\left(3\right)\;\text{If}\;S\subseteq T\;\text{then}\;{\underline{N}}_{j}^{i}\left(S\right)\subseteq {\underline{N}}_{j}^{i}\left(T\right)$.

$\left(4\right)\;\text{If}\;S\subseteq T,\text{then}\;{\bar{N}}_{j}^{i}\left(S\right)\subseteq {\bar{N}}_{j}^{i}\left(T\right)$.

$\left(5\right){\underline{N}}_{j}^{i}\left(S\cap T\right)={\underline{N}}_{j}^{i}\left(S\right)\cap {\underline{N}}_{j}^{i}\left(T\right)$.

$\left(6\right){\bar{N}}_{j}^{i}\left(S\cup T\right)={\bar{N}}_{j}^{i}\left(S\right)\cup {\bar{N}}_{j}^{i}\left(T\right)$.

$\left(7\right)\;{\underline{N}}_{j}^{i}\left(S\cup T\right)⊇{\underline{N}}_{j}^{i}\left(S\right)\cup {\underline{N}}_{j}^{i}\left(T\right)$.

$\left(8\right){\bar{N}}_{j}^{i}\left(S\cap T\right)\subseteq {\bar{N}}_{j}^{i}\left(S\right)\cap {\bar{N}}_{j}^{i}\left(T\right)$.

$\left(9\right)\;{\underline{N}}_{j}^{i}\left(S\right)={\left[{\bar{N}}_{j}^{i}\left(S^{c}\right)\right]}^{c},S^{c}\;\text{is}\;\text{the}\;\text{complement}\;\text{of}\;S$.

$\left(10\right)\;{\bar{N}}_{j}^{i}\left(S\right)={\left[{\underline{N}}_{j}^{i}\left(S^{c}\right)\right]}^{c}$.

$\left(11\right)\;{\underline{N}}_{j}^{i}\left({\underline{N}}_{j}^{i}\left(S\right)\right)={\underline{N}}_{j}^{i}\left(S\right)$.

$\left(12\right)\;{\bar{N}}_{j}^{i}\left({\bar{N}}_{j}^{i}\left(S\right)\right)={\bar{N}}_{j}^{i}\left(S\right)$.

**Proof:** We prove the proposition in the case N=P only, and the other cases similarly.

(1) and (2): These are stated to be obvious by Definition 3.1.

(3) Let S⊆T, then ${\underline{R}}_{j}^{i}\left(S\right)\subseteq {\underline{R}}_{j}^{i}\left(T\right)$ and ${\bar{R}}_{j}^{i}\left(S\right)\subseteq {\bar{R}}_{j}^{i}\left(T\right)$. Thus,

$${\underline{P}}_{j}^{i}\left(S\right)=S\cap {\underline{R}}_{j}^{i}\left({\bar{R}}_{j}^{i}\left(S\right)\right)\subseteq T\cap {\underline{R}}_{j}^{i}\left({\bar{R}}_{j}^{i}\left(T\right)\right)={\underline{P}}_{j}^{i}\left(T\right)\text{.}$$

(4) Follow a similar path as (3).

(5) First, since S∩T⊆S and S∩T⊆T. Then, by (3), ${\underline{P}}_{j}^{i}\left(S\cap T\right)\subseteq {\underline{P}}_{j}^{i}\left(S\right)\cap {\underline{P}}_{j}^{i}\left(T\right)$.

Now, let $w\in \left[{\underline{P}}_{j}^{i}\left(S\right)\cap {\underline{P}}_{j}^{i}\left(T\right)\right]$. Then $w\in {\underline{P}}_{j}^{i}\left(S\right)$ and $w\in {\underline{P}}_{j}^{i}\left(T\right)$ which implies

$w\in \left[S\cap {\underline{R}}_{j}^{i}\left({\bar{R}}_{j}^{i}\left(S\right)\right)\right]$ and $w\in \left[T\cap {\underline{R}}_{j}^{i}\left({\bar{R}}_{j}^{i}\left(T\right)\right)\right]$. Consequently, by Proposition 2.3, $w\in \left[\left(S\cap T\right)\cap {\underline{R}}_{j}^{i}\left({\bar{R}}_{j}^{i}\left(\left(S\cap T\right)\right)\right)\right]={\underline{P}}_{j}^{i}\left(S\cap T\right)$. Thus, ${\underline{P}}_{j}^{i}\left(S\right)\cap {\underline{P}}_{j}^{i}\left(T\right)\subseteq {\underline{P}}_{j}^{i}\left(S\cap T\right)$.

(6) Follow a similar path as (5).

(7) and (8) By using properties (3) and (4), the proof is obvious.

$\left(9\right)\;{\left[{\bar{P}}_{j}^{i}\left(S^{c}\right)\right]}^{c}={\left[S^{c}\cup {\bar{R}}_{j}^{i}\left({\underline{R}}_{j}^{i}\left(S^{c}\right)\right)\right]}^{c}=S\cap {\left[{\bar{R}}_{j}^{i}\left({\underline{R}}_{j}^{i}\left(S^{c}\right)\right)\right]}^{c}$. But, using Proposition 2.3, we get ${\left[{\bar{P}}_{j}^{i}\left(S^{c}\right)\right]}^{c}=S\cap {\underline{R}}_{j}^{i}\left({\bar{R}}_{j}^{i}\left(S\right)\right)={\underline{P}}_{j}^{i}\left(S\right)$.

(10) Follow a similar path as (9).

(11) First, by using (1), we have ${\underline{P}}_{j}^{i}\left({\underline{P}}_{j}^{i}\left(S\right)\right)\subseteq {\underline{P}}_{j}^{i}\left(S\right)$.

Now, let $w\notin {\underline{P}}_{j}^{i}\left({\underline{P}}_{j}^{i}\left(S\right)\right)$. Then $w\notin \left[{\underline{P}}_{j}^{i}\left(S\right)\cap {\underline{R}}_{j}^{i}\left({\bar{R}}_{j}^{i}\left({\underline{P}}_{j}^{i}\left(S\right)\right)\right)\right]$ which means that $w\notin {\underline{P}}_{j}^{i}\left(S\right)$ and $w\notin {\underline{R}}_{j}^{i}\left({\bar{R}}_{j}^{i}\left({\underline{P}}_{j}^{i}\left(S\right)\right)\right)$. Accordingly, $n_{j}^{i}\left(w\right)\cap {\bar{R}}_{j}^{i}\left({\underline{P}}_{j}^{i}\left(S\right)\right)=\emptyset$ and this implies $n_{j}^{i}\left(w\right)⊈{\underline{P}}_{j}^{i}\left(S\right)$. Therefore $w\notin {\underline{P}}_{j}^{i}\left(S\right)$ and hence ${\underline{P}}_{j}^{i}\left(S\right)\subseteq {\underline{P}}_{j}^{i}\left({\underline{P}}_{j}^{i}\left(S\right)\right)$.

(12) Follow a similar path as (11). ∎.

Remark 3.1 It should be noted that the different types of nearly-initial approximations, for each N∈{P,S,γ,α,β}, are independent or comparable. That is, the following relations are not true in general.

$\left(1\right)\;{\underline{N}}_{⋎}^{i}\left(S\right)\subseteq {\underline{N}}_{r}^{i}\left(S\right)\subseteq {\underline{N}}_{⋏}^{i}\left(S\right)$. (2)${\underline{N}}_{⋎}^{i}\left(S\right)\subseteq {\underline{N}}_{l}^{i}\left(S\right)\subseteq {\underline{N}}_{⋏}^{i}\left(S\right)$.

$\left(3\right)\;{\bar{N}}_{⋏}^{i}\left(S\right)\subseteq {\bar{N}}_{r}^{i}\left(S\right)\subseteq {\bar{N}}_{⋎}^{i}\left(S\right)$. (4)${\bar{N}}_{⋏}^{i}\left(S\right)\subseteq {\bar{N}}_{l}^{i}\left(S\right)\subseteq {\bar{N}}_{⋎}^{i}\left(S\right)$.

Examples 3.1 & 3.2 explain this remark in the case of N=P, and the other cases follow a similar pattern.

Example 3.1 Let U={t,u,v,w} and R={(t,t),(t,u),(t,v),(u,t),(u,u),(u,v),(v,v), (w,v), (w,w)}. Thus, we get Table 1 as follows:

**Table 1:** Initial j-neighborhoods of x∈U:

x	$n_{r}^{i}\left(x\right)$	$n_{\;l}^{i}\left(x\right)$	$n_{⋎}^{i}\left(x\right)$
t	{t,u,v}	{t,u}	{t,u,v}
u	{t,u,v}	{t,u}	{t,u,v}
v	{v}	U	U
w	{v,w}	{w}	{v,w}

Now, if W={u,w}, then ${\underline{P}}_{r}^{i}\left(W\right)=\varphi$. But ${\underline{P}}_{⋎}^{i}\left(W\right)=W$, hence ${\underline{P}}_{⋎}^{i}\left(W\right)⊈{\underline{P}}_{r}^{i}\left(W\right)$.

Also, if V={v}, then ${\bar{P}}_{r}^{i}\left(V\right)=U$. But ${\bar{P}}_{⋎}^{i}\left(V\right)=V$, thus ${\bar{P}}_{r}^{i}\left(V\right)⊈{\bar{P}}_{⋎}^{i}\left(V\right)$.

Example 3.2 Let R={(t,t),(t,u),(u,t),(u,u),(v,t),(w,t),(w,w)} be a relation on the set U={t,u,v,w}. Thus, we get Table 2 as follows:

**Table 2:** Initial j-neighborhoods of x∈U:

x	$n_{r}^{i}\left(x\right)$	$n_{\;l}^{i}\left(x\right)$	$n_{⋏}^{i}\left(x\right)$
t	{t,u}	{t}	{t}
u	{t,u}	{t,u}	{t,u}
v	U	U	U
w	{w}	{t,w}	{w}

Now, if W={u,v}, then ${\underline{P}}_{r}^{i}\left(W\right)=\left\{u\right\}$. But ${\underline{P}}_{⋏}^{i}\left(W\right)=\varphi$, hence ${\underline{P}}_{r}^{i}\left(W\right)⊈{\underline{P}}_{⋏}^{i}\left(W\right)$.

Also, if V={t}, then ${\bar{P}}_{r}^{i}\left(V\right)=V$. But ${\bar{P}}_{⋏}^{i}\left(V\right)=\left\{t,u,v\right\}$, thus ${\bar{P}}_{⋏}^{i}\left(V\right)⊈{\bar{P}}_{r}^{i}\left(V\right)$.

The following results introduce a comparison between the different types of nearly-initial approximations.

Proposition 3.2 Let $\left(U,R,\xi_{j}\right)$ be a j**-**NS and S⊆U. Then the following statements hold:

(i) ${\underline{\alpha }}_{j}^{i}\left(S\right)\subseteq {\underline{S}}_{j}^{i}\left(S\right)\subseteq {\underline{\gamma }}_{j}^{i}\left(S\right)\subseteq {\underline{\beta }}_{j}^{i}\left(S\right)$.
(ii) ${\underline{\alpha }}_{j}^{i}\left(S\right)\subseteq {\underline{P}}_{j}^{i}\left(S\right)\subseteq {\underline{\gamma }}_{j}^{i}\left(S\right)\subseteq {\underline{\beta }}_{j}^{i}\left(S\right)$.
(iii) ${\bar{\beta }}_{j}^{i}\left(S\right)\subseteq {\bar{\gamma }}_{j}^{i}\left(S\right)\subseteq {\bar{S}}_{j}^{i}\left(S\right)\subseteq {\bar{\alpha }}_{j}^{i}\left(S\right)$.
(iv) ${\bar{\beta }}_{j}^{i}\left(S\right)\subseteq {\bar{\gamma }}_{j}^{i}\left(S\right)\subseteq {\bar{P}}_{j}^{i}\left(S\right)\subseteq {\bar{\alpha }}_{j}^{i}\left(S\right)$.

**Proof:** We'll prove the first statement; the others follow similarly.

Let $w\in {\underline{\alpha }}_{j}^{i}\left(S\right)$. Then w∈S and $w\in {\underline{R}}_{j}^{i}\left[{\bar{R}}_{j}^{i}\left({\underline{R}}_{j}^{i}\left(S\right)\right)\right]$, implying w∈S and $w\in {\bar{R}}_{j}^{i}\left({\underline{R}}_{j}^{i}\left(S\right)\right)$. Accordingly, $w\in {\underline{S}}_{j}^{i}\left(S\right)$, hence $w\in {\underline{\gamma }}_{j}^{i}\left(S\right)$. Now $w\in {\underline{\gamma }}_{j}^{i}\left(S\right)$ implies $w\in \left[{\underline{P}}_{j}^{i}\left(S\right)\cap {\underline{S}}_{j}^{i}\left(S\right)\right]\subseteq {\underline{P}}_{j}^{i}\left(S\right)$ which means w∈S and $w\in {\underline{R}}_{j}^{i}({\bar{R}}_{j}^{i}\left(S\right)$. Therefore, w∈S and $w\in {\bar{R}}_{j}^{i}\left[{\underline{R}}_{j}^{i}({\bar{R}}_{j}^{i}\left(S\right)\right]$, implying $w\in {\underline{\beta }}_{j}^{i}\left(S\right)$. ∎:

Corollary 3.1 Let $\left(U,R,\xi_{j}\right)$ be a j**-**NS, and S⊆U. Then the following statements hold:

(i) $B_{\beta_{j}}^{i}\left(S\right)\subseteq B_{\gamma_{j}}^{i}\left(S\right)\subseteq B_{S_{j}}^{i}\left(S\right)\subseteq B_{\alpha_{j}}^{i}\left(S\right)$.
(ii) $B_{\beta_{j}}^{i}\left(S\right)\subseteq B_{\gamma_{j}}^{i}\left(S\right)\subseteq B_{P_{j}}^{i}\left(S\right)\subseteq B_{\alpha_{j}}^{i}\left(S\right)$.
(iii) $\mu_{\alpha_{j}}^{i}\left(S\right)\leq \mu_{S_{j}}^{i}\left(S\right)\leq \mu_{\gamma_{j}}^{i}\left(S\right)\leq \mu_{\beta_{j}}^{i}\left(S\right)$.
(iv) $\mu_{\alpha_{j}}^{i}\left(S\right)\leq \mu_{P_{j}}^{i}\left(S\right)\leq \mu_{\gamma_{j}}^{i}\left(S\right)\leq \mu_{\beta_{j}}^{i}\left(S\right)$.
(v) S is $\alpha_{j}^{i}$-exact ⇒ S is $S_{j}^{i}$-exact ⇒ S is $\gamma_{j}^{i}$-exact ⇒ S is $\beta_{j}^{i}$-exact.
(vi) S is $\alpha_{j}^{i}$-exact ⇒ S is $P_{j}^{i}$-exact ⇒ S is $\gamma_{j}^{i}$-exact ⇒ S is $\beta_{j}^{i}$-exact.

Remark 3.2

(i) The converse of the above results is generally not true.
(ii) $S_{j}^{i}$-approximations and $P_{j}^{i}$-approximations are independent.

The subsequent example (Example 3.3) illustrates Remark 3.2 and offers comparisons among various types of nearly-initial approximations.

Example 3.3 Consider U={t,u,v,w} and R={(t,t),(t,u),(t,v),(u,t),(u,u),(u,v), (v,v), (w,v), (w,w)}. Thus, we get $n_{r}^{i}\left(t\right)=n_{r}^{i}\left(u\right)=\left\{t,u\right\}$, $n_{r}^{i}\left(v\right)=U$, and $n_{r}^{i}\left(w\right)=\left\{w\right\}$.

To demonstrate the independence of $S_{j}^{i}$-approximations and $P_{j}^{i}$-approximations: Let S={t,u} and T={t,v,w}. Then ${\underline{S}}_{r}^{i}\left(S\right)={\bar{S}}_{r}^{i}\left(S\right)=S$, implying $B_{S_{r}}^{i}\left(S\right)=\varphi$ and $\mu_{S_{r}}^{i}\left(S\right)=1$, indicating S is $S_{r}^{i}$-exact. But ${\underline{P}}_{j}^{i}\left(S\right)=\{t,u\}$ and ${\bar{P}}_{j}^{i}\left(S\right)=\{t,u,v\}$, implying $B_{P_{r}}^{i}\left(S\right)=\{v\}$, and $\mu_{P_{r}}^{i}\left(S\right)=0.66$, indicating S is $P_{r}^{i}$-rough. On the other hand, ${\underline{P}}_{j}^{i}\left(T\right)={\bar{P}}_{j}^{i}\left(T\right)=T,\text{implying}\;B_{P_{r}}^{i}\left(T\right)=\varphi$ and $\mu_{P_{r}}^{i}\left(T\right)=1$, indicating T is $P_{r}^{i}$-exact. But ${\underline{S}}_{r}^{i}\left(T\right)=\left\{v,w\right\}$ and ${\bar{S}}_{r}^{i}\left(T\right)=U$, implying $B_{S_{r}}^{i}\left(T\right)=\{t,u\}$ and $\mu_{S_{r}}^{i}\left(T\right)=0.5$, indicating T is $S_{r}^{i}$-rough.

Now, we demonstrate that the converse of Proposition 3.2 and Corollary 3.1 is not true by illustrating comparisons among various nearly j-initial accuracy measures ($\mu_{N_{j}}^{i}\left(S\right)$) for all subsets in U, considering different types of N∈{P,S,γ,α,β}. This comparison is depicted in Table 3 for the case of =r , with similar analyses for other j values.

**Table 3:** Comparisons among different types of nearly j-initial accuracy measures.

S⊆U	$\mu_{\alpha_{j}}^{i}\left(S\right)$	$\mu_{P_{j}}^{i}\left(S\right)$	$\mu_{S_{j}}^{i}\left(S\right)$	$\mu_{\gamma_{j}}^{i}\left(S\right)$	$\mu_{\beta_{j}}^{i}\left(S\right)$
{t}	0	1	0	1	1
{u}	0	1	0	1	1
{v}	0	0	0	0	0
{w}	0.5	0.5	1	1	1
{t,u}	0.66	0.66	1	1	1
{t,v}	0	0.5	0	0.5	1
{t,w}	0.25	0.66	0.25	0.66	1
{u,v}	0	0.5	0	0.5	1
{u,w}	0.25	0.66	0.25	0.66	1
{v,w}	0.5	0.5	1	1	1
{t,u,v}	0.66	0.66	1	1	1
{t,u,w}	0.75	0.75	0.75	0.75	0.75
{t,v,w}	0.25	1	0.5	1	1
{u,v,w}	0.25	1	0.5	1	1
U	1	1	1	1	1

**Fig. 1:**
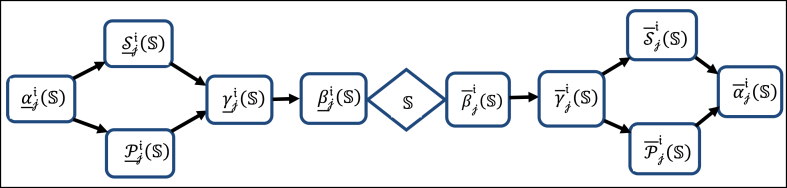
The relationships among different types of nearly approximations

Remark 3.3 Based on Example 3.3, it's evident that the most precise method for approximating rough sets is through the utilization of $\beta_{j}^{i}$-approximations. Additionally, Fig. 1 below illustrates the relationships among various types of approximate sets.

## 4 Comparisons between nearly j-initial approximations and the previous methods

In this section, we present comparisons between the proposed methods and previous proposals, specifically Yao [3], Abd El-Monsef et al. [11], Dai et al. [6], El-Sayed et al. [19], and Abu-Gadairi [39], through established results and counterexamples. Our aim is to demonstrate the superior accuracy of the suggested methods over these prior approaches.

### 4.1 Comparison between El-Sayed et al., Abu-Gdairi techniques and the suggested method

To begin, we introduce Lemma 4.1, highlighting the relationships between the techniques proposed by El-Sayed et al. [19], Abu-Gadairi [40], and the suggested nearly j-initial approximations.

Lemma 4.1 Let $\left(U,R,\xi_{j}\right)$ be a j**-**NS, and S⊆U. Then:

(i) ${\underline{R}}_{j}^{i}\left(S\right)\subseteq {\underline{R}}_{j}^{i}\left({\bar{R}}_{j}^{i}\left(S\right)\right)$.
(ii) ${\bar{R}}_{j}^{i}\left({\underline{R}}_{j}^{i}\left(S\right)\right)\subseteq {\bar{R}}_{j}^{i}\left(S\right)$.

**Proof:** We demonstrate the first item; the second follows similarly.

Utilizing Proposition 2.3, we establish ${\underline{R}}_{j}^{i}\left({\underline{R}}_{j}^{i}\left(S\right)\right)={\underline{R}}_{j}^{i}\left(S\right)$ and ${\underline{R}}_{j}^{i}\left(S\right)\subseteq {\bar{R}}_{j}^{i}\left(S\right)$. Thus ${\underline{R}}_{j}^{i}\left({\underline{R}}_{j}^{i}\left(S\right)\right)\subseteq {\underline{R}}_{j}^{i}\left({\bar{R}}_{j}^{i}\left(S\right)\right)$ which implies ${\underline{R}}_{j}^{i}\left(S\right)\subseteq {\underline{R}}_{j}^{i}\left({\bar{R}}_{j}^{i}\left(S\right)\right)$. ∎:

Theorem 4.1 Consider $\left(U,R,\xi_{j}\right)$ is a j**-**NS and S⊆U. Then, ∀N∈{P,S,γ,α,β}:

(i) ${\underline{R}}_{j}^{i}\left(S\right)\subseteq {\underline{N}}_{j}^{i}\left(S\right)$.
(ii) ${\bar{N}}_{j}^{i}\left(S\right)\subseteq {\bar{R}}_{j}^{i}\left(S\right)$.

**Proof:** We prove the first statement in the case N=P and the others similarly.

Let $w\in {\underline{R}}_{j}^{i}\left(S\right)$, then $n_{j}^{i}\left(w\right)\subseteq S$ such that $w\in n_{j}^{i}\left(w\right)$ which implies w∈S. But, by Lemma 4.1, ${\underline{R}}_{j}^{i}\left(S\right)\subseteq {\underline{R}}_{j}^{i}\left({\bar{R}}_{j}^{i}\left(S\right)\right)$ which means that $w\in {\underline{R}}_{j}^{i}\left({\bar{R}}_{j}^{i}\left(S\right)\right)$. Therefore, $w\in S\cap {\underline{R}}_{j}^{i}\left({\bar{R}}_{j}^{i}\left(S\right)\right)={\underline{P}}_{j}^{i}\left(S\right)$ which implies ${\underline{R}}_{j}^{i}\left(S\right)\subseteq {\underline{P}}_{j}^{i}\left(S\right)$. ∎:

Corollary 4.1 Consider $\left(U,R,\xi_{j}\right)$ is a j**-**NS and S⊆U. Then, ∀N∈{P,S,γ,α,β}:

(i) $B_{N_{j}}^{i}\left(S\right)\subseteq B_{j}^{i}\left(S\right)$ .
(ii) $\mu_{j}^{i}\left(S\right)\leq \mu_{N_{j}}^{i}\left(S\right)$.
(iii) The subset S is an j-initial exact set if it is $N_{j}^{i}$-initial exact.

Remark 4.1 According to Theorem 4.1 and Corollary 4.1, the methods proposed in Definition 3.1 and Definition 3.2 represent generalizations of the techniques introduced by El-Sayed et al. [19] and Abu-Gadairi [39]. In other words, the nearly j-initial approaches demonstrate greater accuracy compared to them. However, the converse of these results is not universally true, as confirmed by Example 4.1 and Example 4.2.

To illustrate this, we note that El-Sayed et al.'s approach represents a specific case within Abu-Gdairi's techniques when j=r. Hence, we compute the approximations using j-initial neighborhoods in the case of j=r, specifically comparing Abu-Gadairi's technique and the suggested method, as elucidated in Example 4.1.

Example 4.1 Consider U={t,u,v,w} and R={(t,t),(t,u),(u,t),(u,u),(v,t),(w,t), (w,w)} as a binary relation on U. Consequently, we have $n_{r}^{i}\left(t\right)=n_{r}^{i}\left(u\right)=\left\{t,u\right\}$, $n_{r}^{i}\left(v\right)=U$, and $n_{r}^{i}\left(w\right)=\left\{w\right\}$. To assess the accuracy of approximations, we compare the results obtained using El-Sayed et al. [19] (Abu-Gadairi [39], in the case of j=r) technique with the suggested method ($\beta_{r}$-approximations), as detailed in Table 4.

**Table 4:** Comparison of accuracy measures between El-Sayed et al. [19] (Abu-Gdairi technique [40], in the case of j=r) and the current method ($\beta_{r}$-accuracy) in the case of j=r:

S⊆U	El-Sayed et al.	Current method
	$\mu_{r}\left(S\right)$	$\mu_{\beta_{r}}^{i}\left(S\right)$
{t}	0	1
{u}	0	1
{v}	0	0
{w}	0.5	1
{t,u}	0.66	1
{t,v}	0	1
{t,w}	0.25	1
{u,v}	0	1
{u,w}	0.25	1
{v,w}	0.5	1
{t,u,v}	0.66	1
{t,u,w}	0.75	0.75
{t,v,w}	0.25	1
{u,v,w}	0.25	1
U	1	1

Continuing from Example 4.1, when evaluating the approximations using j-initial neighborhoods in the case of j=⋏, we compare Abu-Gadairi's [39] technique with the suggested method.

**Example 4.2.** Using the same U={t,u,v,w} and R={(t,t),(t,u),(u,t),(u,u),(v,t), (w,t), (w,w)} as previously described, the j-initial neighborhoods for j=⋏ are determined as follows: $n_{⋏}^{i}\left(t\right)=\left\{t\right\}$, $n_{⋏}^{i}\left(u\right)=\left\{t,u\right\}$, $n_{⋏}^{i}\left(v\right)=U$, and $n_{⋏}^{i}\left(w\right)=\left\{w\right\}$. Let's consider S={t,u,v}, hence ${\underline{R}}_{⋏}^{i}\left(S\right)=\left\{t,u\right\}$ and ${\bar{R}}_{⋏}^{i}\left(S\right)=\left\{t,u,v\right\}$. Consequently, $B_{⋏}^{i}\left(S\right)=\{v\}$, resulting in $\mu_{⋏}^{i}\left(S\right)=0.66$, indicating that S is ⋏-rough as per Abu-Gadairi's technique. On the other hand, ${\underline{\beta }}_{⋏}^{i}\left(S\right)={\bar{\beta }}_{⋏}^{i}\left(S\right)=S$, leading to $B_{\beta_{⋏}}^{i}\left(S\right)=\varphi$ and $\mu_{\beta_{⋏}}^{i}\left(S\right)=1$. Therefore, S qualifies as a $\beta_{⋏}^{i}$-exact set. By applying a similar approach to other subsets of U, it becomes evident that the $\beta_{⋏}^{i}$-approach method is consistently more accurate than Abu-Gadairi's techniques.

### 4.2 Comparison of the suggested method versus the technique of Dai et al

Initially, in the broader context of binary relations, the initial approximations and Dai et al.'s rough sets [6] (maximal rough approximations) stand as independent entities, as detailed in the paper [19]. Consequently, maximal approximations and nearly initial approximations maintain their independence in this scenario. However, it's crucial to note that Dai et al.'s method's approximations do not adhere to the fundamental properties of Pawlak theory. In contrast, the suggested approach (nearly initial approximations) fulfills all the axioms of Pawlak principles in the general case without imposing any limitations (refer to Proposition 3.1).

Subsequently, the forthcoming results and exemplars elucidate the interconnections between the proposed methods and Yao rough sets, particularly within the confines of a specialized case within binary relations.

Theorem 4.2 [[19], [39]] If $\left(U,R,\xi_{j}\right)$ represents a j**-**NS and S⊆U within a reflexive relation on U, then:

(i) ${\underline{R}}_{m}\left(S\right)\subseteq {\underline{R}}_{j}^{i}\left(S\right)\subseteq S\subseteq {\bar{R}}_{j}^{i}\left(S\right)\subseteq {\bar{R}}_{m}\left(S\right)$.
(ii) $B_{j}^{i}\left(S\right)\subseteq B_{m}\left(S\right)$, and $\mu_{m}\left(S\right)\leq \mu_{j}^{i}\left(S\right)$.
(iii) If S is m-exact, then S is j-initial exact.

Theorem 4.3 For $\left(U,R,\xi_{j}\right)$ as a j**-**NS and S⊆U within a reflexive relation on , considering ∀N∈{P,S,γ,α,β}:

(i) ${\underline{R}}_{m}\left(S\right)\subseteq {\underline{R}}_{j}^{i}\left(S\right)\subseteq {\underline{N}}_{j}^{i}\left(S\right)\subseteq S\subseteq {\bar{N}}_{j}^{i}\left(S\right)\subseteq {\bar{R}}_{j}^{i}\left(S\right)\subseteq {\bar{R}}_{m}\left(S\right)$.
(ii) $B_{j}^{i}\left(S\right)\subseteq B_{j}\left(S\right)\subseteq B_{m}\left(S\right)$ and $\mu_{m}\left(S\right)\leq \mu_{j}^{i}\left(S\right)\leq \mu_{N_{j}}^{i}\left(S\right)$.
(iii) S is m-exact ⇒ S is j-initial exact ⇒ S is $N_{j}^{i}$-exact.

**proof:** The proof becomes evident by employing Theorems 4.1 and 4.2. ∎.

Remark 4.2 It's essential to note that the inverse of the aforementioned results isn't generally valid, as demonstrated in Example 4.3.

Example 4.3 If U={t,u,v,w} and R={(t,t),(t,u),(u,u),(u,v),(v,v), (w,w)} is a reflexive relation on U. Thus, the r-initial neighborhoods are:

$$n_{r}^{i}\left(t\right)=\left\{t,u\right\},n_{r}^{i}\left(u\right)=\left\{u\right\},n_{r}^{i}\left(v\right)=\left\{u,v\right\},\text{and}\;n_{r}^{i}\left(w\right)=\left\{w\right\}\text{.}$$

Also, the m-neighborhoods are:

$$n_{m}\left(t\right)=\left\{t,u\right\},n_{m}\left(u\right)=\left\{t,u,v\right\},n_{m}\left(v\right)=\left\{u,v\right\},\text{and}\;n_{m}\left(w\right)=\left\{w\right\}\text{.}$$

So, we compare between the previous work (m-approximations [6]), and the suggested method ($\beta_{j}^{i}$-approximations) as Table 5 explains.

**Table 5:** Comparisons among the accuracy measures of Dai et al. [6], and current methods.

S⊆U	Dai et al.	Current method
	$\mu_{m}\left(S\right)$	$\mu_{\beta_{r}}^{i}\left(S\right)$
{t}	0	1
{u}	0	0.5
{v}	0	0
{w}	1	1
{t,u}	0.33	0.66
{t,v}	0	0.5
{t,w}	0.5	1
{u,v}	0.66	1
{u,w}	0.5	0.66
{v,w}	0.5	0.5
{t,u,v}	1	1
{t,u,w}	0.75	0.75
{t,v,w}	0.33	1
{u,v,w}	0.75	1
U	1	1

### 4.3 Comparison between Yao method and the proposed technique

In the broader context of binary relations, the independence between initial approximations and Yao rough sets is established, as expounded in reference [19]. Consequently, within this scope, Yao's rough sets and nearly initial approximations remain distinct. However, it's crucial to note that Yao's approximations fail to adhere to the foundational principles of Pawlak's theory, as evidenced by Yao [3] and Example 2.2. In contrast, the suggested methodology based on nearly initial approximations rigorously satisfies all axioms of Pawlak principles without imposing any constraints (refer to Proposition 3.1).

The subsequent outcomes and exemplars will elucidate the correlations between the proposed techniques and Yao rough sets, particularly in a specialized instance within binary relations.

Lemma 4.2 [19] For a reflexive and symmetric relation R on U, ∀x∈U: $n_{r}^{i}\left(x\right)\subseteq n_{r}\left(x\right)$.

Theorem 4.4 Given $\left(U,R,\xi_{j}\right)$ as a j**-**NS and S⊆U, where R is a reflexive and symmetric relation on U , the following holds:

(i) $\underline{R}\left(S\right)\subseteq {\underline{R}}_{r}^{i}\left(S\right)\subseteq S\subseteq {\bar{R}}_{r}^{i}\left(S\right)\subseteq \bar{R}\left(S\right)$.
(ii) $B_{r}^{i}\left(S\right)\subseteq B\left(S\right)$ and $\mu \left(S\right)\leq \mu_{r}^{i}\left(S\right)$.
(iii) If S is a Yao-exact set, then it is j-initial exact.

**Proof:** We'll establish the validity of the first statement; subsequently, the others will follow using a similar approach.

Let $x\in \underline{R}\left(S\right)$, implying $n_{r}\left(x\right)\subseteq S$. Thus, according to Lemma 4.2, $n_{r}^{i}\left(x\right)\subseteq S$, implying $x\in {\underline{R}}_{r}^{i}\left(S\right)$. Therefore, $\underline{R}\left(S\right)\subseteq {\underline{R}}_{r}^{i}\left(S\right)$ and likewise, applying the same logic, we deduce ${\bar{R}}_{r}^{i}\left(S\right)\subseteq \bar{R}\left(S\right)$. ∎:

Theorem 4.5 Let $\left(U,R,\xi_{j}\right)$ be a j**-**NS and S⊆U, where R is a reflexive and symmetric relation on U. Then, ∀N∈{P,S,γ,α,β}:

(i) $\underline{R}\left(S\right)\subseteq {\underline{R}}_{j}^{i}\left(S\right)\subseteq {\underline{N}}_{j}^{i}\left(S\right)$.
(ii) ${\bar{N}}_{j}^{i}\left(S\right)\subseteq {\bar{R}}_{j}^{i}\left(S\right)\subseteq \bar{R}\left(S\right)$.
(iii) $B_{N_{j}}^{i}\left(S\right)\subseteq B_{j}^{i}\left(S\right)\subseteq B\left(S\right)$.
(iv) $\mu \left(S\right)\leq \mu_{j}^{i}\left(S\right)\leq \mu_{N_{j}}^{i}\left(S\right)$.
(v) The subset S is a j-exact set ⇒ S is j-initial exact ⇒ S is $N_{j}^{i}$-exact.

**proof:** The proof is evident by employing Theorem 4.1 and Theorem 4.4. ∎:

Remark 4.3 The converse of the aforementioned results does not hold generally, as confirmed in Example 4.4.

Example 4.4 Given U={t,u,v,w} and R={(t,t),(t,u),(u,u),(u,v),(v,v),(w,w)} as a reflexive and symmetric relation on U. Thus, we obtain:

$$n_{r}\left(t\right)=\left\{t,u\right\},n_{r}\left(u\right)=\left\{t,u,v\right\},n_{r}\left(v\right)=\left\{u,v\right\},\text{and}\;n_{r}\left(w\right)=\left\{w\right\}\text{.}$$

Consequently, we derive: $n_{r}^{i}\left(t\right)=\left\{t,u\right\}\text{,}$ $n_{r}^{i}\left(u\right)=\left\{u\right\}$, $n_{r}^{i}\left(v\right)=\left\{u,v\right\}$, and $n_{r}^{i}\left(w\right)=\left\{w\right\}$.

A comparison between Yao's approach [3] and the current method ($\beta_{j}^{i}$-approximations) is illustrated in Table 6.

**Table 6:** Comparisons among accuracy measures of Yao [3] approach and the existing method.

S⊆U	Yao approach	Current method
	μ(S)	$\mu_{\beta_{r}}^{i}\left(S\right)$
{t}	0	0
{u}	0	0.33
{v}	0	0
{w}	1	1
{t,u}	0.33	0.66
{t,v}	0	0
{t,w}	0.33	0.5
{u,v}	0.33	0.66
{u,w}	0.25	0.5
{v,w}	0.33	0.5
{t,u,v}	1	1
{t,u,w}	0.66	0.75
{t,v,w}	0.25	0.33
{u,v,w}	0.5	0.75
U	1	1

### 4.4 Comparison between the methods of Abd El-Monsef et al. And the proposed technique

Theorem 4.6 [19] For $\left(U,R,\xi_{j}\right)$ as a j**-**NS and S⊆U. If R is a reflexive and symmetric relation on U. Then:

(i) ${\underline{R}}_{j}\left(S\right)\subseteq {\underline{R}}_{j}^{i}\left(S\right)\subseteq S\subseteq {\bar{R}}_{j}^{i}\left(S\right)\subseteq {\bar{R}}_{j}\left(S\right)$.
(ii) $B_{j}^{i}\left(S\right)\subseteq B_{j}\left(S\right)$ and $\mu_{j}\left(S\right)\leq \mu_{j}^{i}\left(S\right)$.
(iii) The subset S, being a j-exact set, implies it is also j-initial exact.

Theorem 4.7 Consider $\left(U,R,\xi_{j}\right)$ is a j**-**NS and S⊆U, where R is a reflexive and symmetric relation on U, then ∀N∈{P,S,γ,α,β}:

(i) ${\underline{R}}_{j}\left(S\right)\subseteq {\underline{R}}_{j}^{i}\left(S\right)\subseteq {\underline{N}}_{j}^{i}\left(S\right)$.
(ii) ${\bar{N}}_{j}^{i}\left(S\right)\subseteq {\bar{R}}_{j}^{i}\left(S\right)\subseteq {\bar{R}}_{j}\left(S\right)$.
(iii) $B_{N_{j}}^{i}\left(S\right)\subseteq B_{j}^{i}\left(S\right)\subseteq B_{j}\left(S\right)$.
(iv) $\mu_{j}\left(S\right)\leq \mu_{j}^{i}\left(S\right)\leq \mu_{N_{j}}^{i}\left(S\right)$.
(v) The subset S is a j-exact set ⇒ S is j-initial exact ⇒ S is also $N_{j}^{i}$-exact.

**proof:** The proof is self-evident using Theorem 4.1 and Theorem 4.6. ∎:

Remark 4.4 The converse of the above results isn't generally valid, as demonstrated in Example 4.5.

Example 4.5 (Continuation from Example 4.4) Obtaining $T_{r}=F_{r}=\left\{U,\varphi ,\left\{w\right\},\left\{t,u,v\right\}\right\}$.

A comparison between prior work (Abd El-Monsef et al. [11]) and the suggested method ($\beta_{j}^{i}$-approximations) is depicted in Table 7.

**Table 7:** Comparison among accuracy measures of Abd El-Monsef et al. [11] techniques and the proposed method

S⊆U	Abd El-Monsef et al. techniques	Current method
	$\mu_{r}\left(S\right)$	$\mu_{\beta_{r}}^{i}\left(S\right)$
{t}	0	0
{u}	0	0.33
{v}	0	0
{w}	1	1
{t,u}	0	0.66
{t,v}	0	0
{t,w}	0.25	0.5
{u,v}	0	0.66
{u,w}	0.25	0.5
{v,w}	0.25	0.5
{t,u,v}	1	1
{t,u,w}	0.25	0.75
{t,v,w}	0.25	0.33
{u,v,w}	0.25	0.75
U	1	1

**Concluding remark:** Through the comparisons, the following observations are concluded.

(i) The proposed approach ($\beta_{j}^{i}$-approximations) demonstrates higher accuracy in approximating rough sets compared to previous methods (e.g., Yao [3], Abd El-Monsef et al. [11], Dai et al. [6], El-Sayed et al. [19], and Abu-Gadairi [39]). This is illustrated by the proven results (Theorem 4.1, Theorem 4.2, Theorem 4.3, Theorem 4.4, Theorem 4.5, Theorem 4.6, Theorem 4.7) and illustrative examples (Example 4.1, Example 4.3, Example 4.4, Example 4.5), establishing its utility in discerning roughness and exactness.
(ii) Contrarily, the provided techniques maintain Pawlak's properties of approximation operators, which are absent in certain previous approaches (e.g., Yao and Dai et al.) within the general context of binary relations. For instance, using Yao's approach, inconsistencies arise in specific cases, highlighting the precision of $\beta_{j}^{i}$-approximations approximations in measuring exactness and roughness in general binary relaion, for instance:

Consider the binary relation R={(t,t),(t,u),(u,t),(u,u),(w,t),(w,w)} on the finite set U={t,u,v,w}, and let M={u,w}. Utilizing Yao's approach yields $\bar{R}\left(U\right)=\left\{t,u,w\right\}\neq U$ and $\underline{R}\left(\varphi \right)=\left\{v\right\}\neq \varphi$. Additionally, $\underline{R}\left(M\right)=\left\{v\right\}$ and $\bar{R}\left(M\right)=\left\{t,u,w\right\}$, implying $\underline{R}\left(M\right)⊈M⊈\bar{R}\left(M\right)$. Applying Dai et al.'s approach results in ${\bar{R}}_{m}=\left\{t,u,w\right\}\neq U$ and ${\underline{R}}_{m}\left(\varphi \right)=\left\{v\right\}\neq \varphi$. Also, ${\underline{R}}_{m}\left(M\right)=\left\{v\right\}$, and ${\bar{R}}_{m}\left(M\right)=\left\{t,u,w\right\}$, leading to ${\underline{R}}_{m}\left(M\right)⊈M⊈{\bar{R}}_{m}\left(M\right)$. In contrast, ${\underline{\beta }}_{r}^{i}\left(U\right)$ $={\bar{\beta }}_{r}^{i}\left(U\right)=U$, ${\underline{\beta }}_{r}^{i}\left(\varphi \right)={\bar{\beta }}_{r}^{i}\left(\varphi \right)=\varphi$, and ${\underline{\beta }}_{r}^{i}\left(M\right)={\bar{\beta }}_{r}^{i}\left(M\right)=M$. Consequently, it's evident that $\beta_{j}^{i}$-approximations serve as precise tools for assessing the exactness and roughness of sets.

(iii) Moreover, the current paradigm relaxes certain modeling constraints, allowing a broader space for problem representation, especially in handling larger datasets. This flexibility extends the applicability to analyze and describe various practical problems, including infectious diseases like COVID-19, where the sample size directly impacts accuracy in decision-making as demonstrated in the following section.

## 5 Accurate decision-making in diagnosing COVID-19 variants using nearly initial rough sets

The COVID-19 pandemic has wrought havoc worldwide since January 2020. Despite its various mutations, the most recent strain is proving to be more lethal to humans. The increasing number of active and fatal cases globally and within our country significantly impacts people's psychological well-being. Middle Eastern countries have reported several variants, including Alpha, Delta, and Omicron, each sharing common symptoms alongside more extensive ones. In this section, we propose a framework of rules to categorize and predict COVID-19 variants using provided methodologies, specifically nearly initial-rough sets. These mathematical concepts aid in making accurate decisions and detecting hidden data patterns, potentially saving time and resources for both doctors and patients. Implementing these methods in the decision table of an information system (as described in Ref. [38]), we demonstrate their effectiveness in analyzing the results presented in Table 8.

**Table 8:** [38] Information system of patients infected with COVID variants.

Person	Common symptoms			Extended symptoms					COVID-19 Decision
	FE	LoT/S	FA	BA	LBP	NS	SN	Lo/A	
$p_{1}$	High	No	Yes	Yes	No	Yes	No	Yes	Alpha
$p_{2}$	High	No	Yes	Yes	Yes	Yes	No	Yes	Omicron
$p_{3}$	High	No	No	Yes	No	No	No	No	No
$p_{4}$	Normal	No	No	No	No	No	Yes	No	No
$p_{5}$	Normal	Yes	No	Yes	No	Yes	No	Yes	Delta
$p_{6}$	High	Yes	No	Yes	No	No	No	No	Delta
$p_{7}$	High	Yes	Yes	Yes	No	No	No	No	Delta
$p_{8}$	Normal	No	No	Yes	No	No	No	No	No
$p_{9}$	High	No	No	Yes	Yes	Yes	No	Yes	Omicron
$p_{10}$	High	Yes	Yes	Yes	No	No	No	No	Delta

### 5.1 Experimental results and doctors’ decision table

In this subsection, we present experimental results derived from a preparatory study involving initially ten patients (Table 8), later reduced to eight (Table 9), due to their shared symptoms. Our analysis relied on pre-existing data, with the data source referenced in Chapter [38].

**Table 9:** Information system of patients infected with COVID variants.

Person	symptoms								COVID-19 Decision
	$a_{1}$	$a_{2}$	$a_{3}$	$a_{4}$	$a_{5}$	$a_{6}$	$a_{7}$	$a_{8}$	
$p_{1}$	High	No	Yes	Yes	No	Yes	No	Yes	Alpha
$p_{2}$	High	No	Yes	Yes	Yes	Yes	No	Yes	Omicron
$p_{3}$	High	No	No	Yes	No	No	No	No	No
$p_{4}$	Normal	No	No	No	No	No	Yes	No	No
$p_{5}$	Normal	Yes	No	Yes	No	Yes	No	Yes	Delta
$p_{6}$	High	Yes	No	Yes	No	No	No	No	Delta
$p_{7}$	High	Yes	Yes	Yes	No	No	No	No	Delta
$p_{8}$	Normal	No	No	Yes	No	No	No	No	No

Table 8 showcases the outcomes of 8 symptoms related to COVID-19 variants (Alpha, Delta, or Omicron) across ten patients. The symptoms for each variant—Alpha, Delta, and Omicron—are as follows.

• **Alpha variant:** fever (FE), shortness of breath (SB), body pain (BP), dry cough (DC), headache (HE), sore throat (ST), and chest pain (CP).
• **Delta variant:** FE, cough (CO), SB, BP, CP, ST, HE, loss of taste (LoT), loss of smell (LoS), myalgias (MY), fatigue (FA), and rhinorrhea (RH).
• **Omicron variant:** body ache (BA), weakness (WE), FA, HE, FE, CO, cold (CL), lower back pain (LBP), night sweats (NS), sneezing (SN), and loss of appetite (LoA).

Table 8 summarizes the information about patients infected with either the Alpha, Delta, or Omicron variant. Notably, the Omicron variant shares some common symptoms (FE, CO, SB, FA, LoT, LoS, ST, and HE) with the Alpha and Delta variants, while introducing new symptoms (BA, LBP, NS, SN, and LoA).

We note that the patients $p_{2}$, and $p_{9}$ (resp. $p_{7}$, and $p_{10}$) are similar, so we omit them and hence we obtain the new information system in Table 9.

### 5.2 Mathematical models for decision making of COVID-19 variants

We initiate this application by converting the attributes of symptoms (conditions), denoted as $C=\{a_{1},a_{2\;},a_{3},a_{4},a_{5},a_{6},a_{7},a_{8}\}$ into qualitative terms. Table 9 illustrates the expression of symptom similarities among patients, where the degree of similarity, denoted as ψ(x,y), is determined using the formula:

$$\psi \;\left(x,y\right)=\frac{\sum_{g=1}^{h}\left[a_{g}\left(x\right)=a_{g}\left(y\right)\right]}{h}\text{,}$$

where h represents the total number of condition attributes.

Therefore, Table 10 demonstrates the similarity assessment among patients based on their condition attributes.

**Table 10:** Similarities among symptoms of 8 of patients.

	$p_{1}$	$p_{2}$	$p_{3}$	$p_{4}$	$p_{5}$	$p_{6}$	$p_{7}$	$p_{8}$
$p_{1}$	1	0.875	0.625	0.25	0.625	0.5	0.625	0.5
$p_{2}$	0.875	1	0.5	0.125	0.5	0.375	0.5	0.375
$p_{3}$	0.625	0.5	1	0.625	0.5	0.875	0.75	0.875
$p_{4}$	0.25	0.125	0.5	1	0.375	0.5	0.375	0.75
$p_{5}$	0.625	0.5	0.5	0.375	1	0.625	0.5	0.625
$p_{6}$	0.5	0.375	0.875	0.5	0.625	1	0.875	0.75
$p_{7}$	0.625	0.5	0.75	0.375	0.5	0.875	1	0.625
$p_{8}$	0.5	0.375	0.875	0.75	0.625	0.625	0.75	1

From Table 9, the universe U is divided into two independent sets:

1. The set of individuals **infected** with COVID-19: $S=\left\{p_{1},p_{2},p_{5},p_{6},p_{7}\right\}$.
2. The set of individuals **not infected** with COVID-19: $T=\left\{p_{3},p_{4},p_{8}\right\}$.

Additionally, within the set of COVID-19-infected individuals, further divisions exist.

- Infected persons with the **Omicron** variant: $S_{1}=\left\{p_{1}\right\}$.
- Infected persons with the **Alpha** variant: $S_{2}=\left\{p_{2}\right\}$.
- Infected persons with the **Delta** variant: $S_{3}=\left\{p_{5},p_{6},p_{7}\right\}$.

The subsequent process involves establishing relationships, tailored to the system's requirements. We construct the right neighborhoods and initial-right neighborhoods for each patient in the universe (as illustrated in Table 11) using a relation corresponding to the problem's nature. As per expert insights, we define the relation for each problem as follows: (x,y)∈R⇔ψ(x,y)≥0.75.

**Table 11:** r-neighborhoods, m-neighborhoods, and r-initial neighborhoods of each patient.

x	$n_{r}\left(x\right)$	$n_{m}\left(x\right)$	$n_{r}^{i}\left(x\right)$
$p_{1}$	$\left\{p_{1},p_{2}\right\}$	$\left\{p_{1},p_{2}\right\}$	$\left\{p_{1},p_{2}\right\}$
$p_{2}$	$\left\{p_{1},p_{2}\right\}$	$\left\{p_{1},p_{2}\right\}$	$\left\{p_{1},p_{2}\right\}$
$p_{3}$	$\left\{p_{3},p_{6},p_{7},p_{8}\right\}$	$\left\{p_{3},p_{4},p_{6},p_{7},p_{8}\right\}$	$\left\{p_{3},p_{6}\right\}$
$p_{4}$	$\left\{p_{4},p_{8}\right\}$	$\left\{p_{3},p_{4},p_{6},p_{8}\right\}$	$\left\{p_{4},p_{8}\right\}$
$p_{5}$	$\left\{p_{5}\right\}$	$\left\{p_{5}\right\}$	$\left\{p_{5}\right\}$
$p_{6}$	$\left\{p_{3},p_{6},p_{7},p_{8}\right\}$	$\left\{p_{3},p_{4},p_{6},p_{7},p_{8}\right\}$	$\left\{p_{3},p_{6}\right\}$
$p_{7}$	$\left\{p_{3},p_{6},p_{7}\right\}$	$\left\{p_{3},p_{6},p_{7},p_{8}\right\}$	$\left\{p_{3},p_{6},p_{7}\right\}$
$p_{8}$	$\left\{p_{3},p_{4},p_{6},p_{8}\right\}$	$\left\{p_{3},p_{4},p_{6},p_{7},p_{8}\right\}$	$\left\{p_{8}\right\}$

It's important to note that this relation, symbolized by the number 0.75, signifies the degree of similarity, with higher values indicating increased similarity and more accurate outcomes. This relation and its threshold can be adjusted based on the system experts' perspectives. The advised relation is reflexive and symmetric but lacks transitivity, rendering Pawlak's classification space inadequate for describing this system.

Therefore, we proceed to compute the approximations and their accuracies for the subsets T, S, $S_{1}$, $S_{2}$, and $S_{3}$ using the proposed method ($\beta_{j}^{i}$-approximations). Additionally, we compare these results with previous methodologies presented in works such as Yao [3], Dai et al. [6], El-Sayed et al. [19], and Abu-Gadairi [39]. This comparison aims to highlight the significance of the proposed methods in the medical diagnosis of COVID-19 variants, as demonstrated in Table 12.

**Table 12:** Comparisons of techniques (Yao [3], Dai et al. [6], El-Sayed et al. [19], Abu-Gadairi [39]), and the current method ($\beta_{j}^{i}$-approaches).

M	Yao method			Dai et al. method		
	$\underline{R}\left(M\right)$	$\bar{R}\left(M\right)$	μ(M)	${\underline{R}}_{m}\left(M\right)$	${\bar{R}}_{m}\left(M\right)$	$\mu_{m}\left(M\right)$
T	$\left\{p_{4}\right\}$	$U-\left\{p_{1},p_{2},p_{5}\right\}$	20%	φ	$U-\left\{p_{1},p_{2},p_{5}\right\}$	20%
S	$\left\{p_{1},p_{2},p_{5}\right\}$	$U-\left\{p_{4}\right\}$	42%	$\left\{p_{1},p_{2},p_{5}\right\}$	U	38 %
${\;S}_{1}$	φ	$\left\{p_{1},p_{2}\right\}$	0	φ	$\left\{p_{1},p_{2}\right\}$	0
$S_{2}$	φ	$\left\{p_{1},p_{2}\right\}$	0	φ	$\left\{p_{1},p_{2}\right\}$	0
$S_{3}$	$\left\{p_{5}\right\}$	$U-\left\{p_{1},p_{2},p_{4}\right\}$	20%	$\left\{p_{5}\right\}$	$U-\left\{p_{1},p_{2}\right\}$	16%
M	**El-Sayed et al., and Abu-Gdairi methods**			**Current technique**		
	${\underline{R}}_{r}^{i}\left(M\right)$	${\bar{R}}_{r}^{i}\left(M\right)$	$\mu_{r}^{i}\left(M\right)$	${\underline{\beta }}_{r}^{i}\left(M\right)$	${\bar{\beta }}_{r}^{i}\left(M\right)$	$\mu_{\beta_{j}}^{i}\left(M\right)$
T	$\{\;p_{4},p_{8}\}$	$U-\left\{p_{1},p_{2},p_{5}\right\}$	40%	T	T	100%
S	$\left\{p_{1},p_{2},p_{5}\right\}$	$U-\left\{p_{4},p_{8}\right\}$	50%	S	S	100%
$S_{1}$	φ	$\left\{p_{1},p_{2}\right\}$	0	$S_{1}$	$S_{1}$	100%
$S_{2}$	φ	$\left\{p_{1},p_{2}\right\}$	0	$S_{2}$	$S_{2}$	100%
$S_{3}$	$\left\{p_{5}\right\}$	$\left\{p_{3},p_{5},p_{6},p_{7}\right\}$	25%	$S_{3}$	$S_{3}$	100%

## 6 Discussions

From the previous table (namely, Table 12), we note that.

1) The proposed approaches showcased high accuracy coefficients, aligning mathematically with the medical data from the decision table (Table 9) with 100 % accuracy.
2) Compared to previous methodologies (Yao [3], Dai et al. [6], El-Sayed et al. [19], and Abu-Gadairi [39]), the proposed approaches demonstrated superior accuracy.
3) Earlier methods failed to accurately identify COVID-19 infections and variants despite prior knowledge from the decision table.
4) The provided approaches enhance approximation operators and accuracy measures, extending Pawlak principles to practical problems without additional conditions.
5) These methods utilize induced topological structures, benefiting topological applications in rough set models and their extensions. They're user-friendly, even for non-topology specialists. Presenting topological approximations independently enables establishing rough approximations (initial-approximations) without relying on complex concepts. This simplifies rough-set theories, making them accessible to researchers without extensive topology backgrounds. Democratizing these tools broadens their use. While topology-based methods excel in specialized algorithm development, integrating topological principles into specific algorithms optimizes practical-use algorithms.
6) An algorithm detailed in this paper could be implemented using MATLAB, aiding medical professionals in diagnosing COVID-19. This program could determine infection status and the specific variants (Omicron, Delta, and Alpha) with high accuracy, facilitating appropriate medical procedures for patients.

**Fig. 2:**
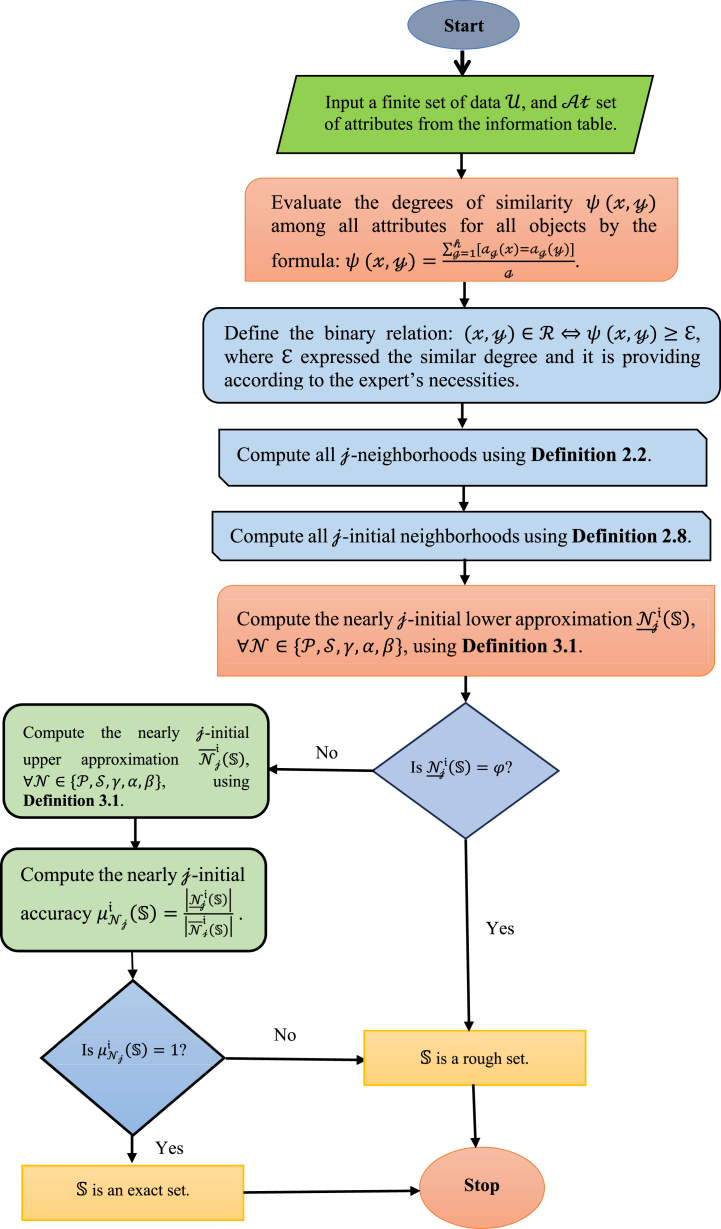
Flowchart to use nearly j-initial approximations in decision-making problems.

Finally, we provide an algorithm and a flowchart of the suggested techniques (nearly j-initial approximations) for helping in decision-making problems. This algorithm (Algorithm 1 and Fig. 2) may be a simple tool that can be used in MATLAB.

**Algorithm 1:**
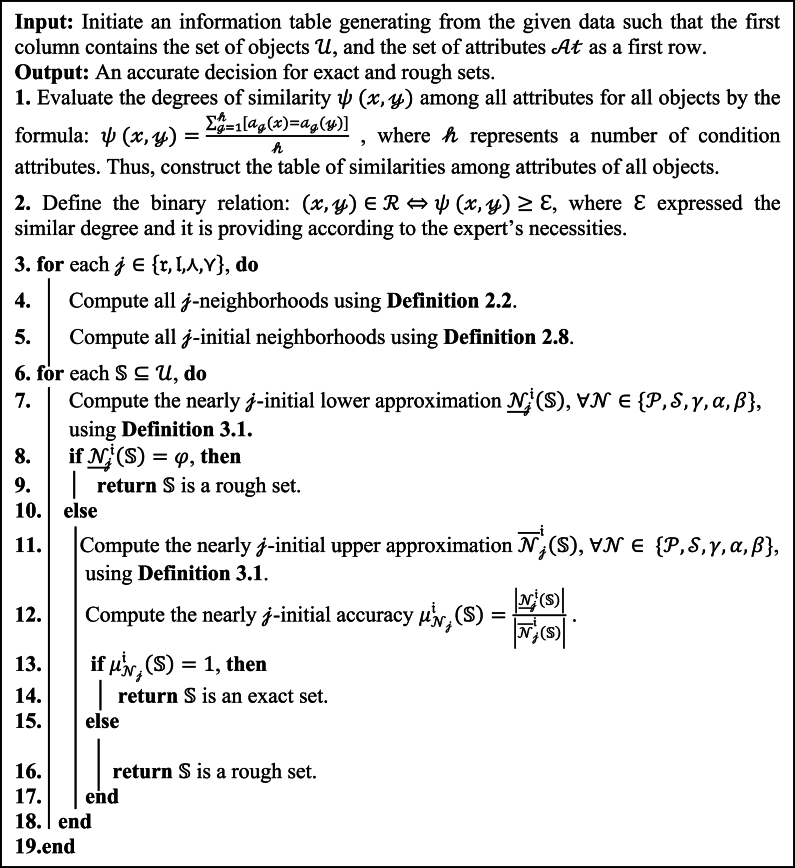
A frame work to use nearly j-initial approximations in decision-making problems

## 7 Conclusion and future work

Initially, we introduced and explored novel extensions to generalized rough sets, referred to as nearly j-initial approximations, rooted in topological structures. These techniques, derived directly from neighborhoods and independent of topology, offer practicality even for non-specialists in topology. We presented five distinct rough approximations, extending Pawlak's rough models and their variations. These approaches notably enhance approximation operators and accuracy measures under any binary relation, outperforming prior methods. Additionally, they maintain Pawlak's principles without conditions (see Proposition 3.1), widening their application scope to diverse practical problems. Comparisons with prior techniques (Yao [3], Abd El-Monsef et al. [11], Dai et al. [6], El-Sayed et al. [19], and Abu-Gadairi [39]) demonstrate the superior accuracy of our proposed methods, as evidenced by proven results (Theorem 4.1, Theorem 4.2, Theorem 4.3, Theorem 4.4, Theorem 4.5, Theorem 4.6, Theorem 4.7) and illustrative examples (Example 4.1, Example 4.3, Example 4.4, Example 4.5). Consequently, these methods offer substantial value in revealing both roughness and exactness.

### 7.1 Advantages of current methods

1) **Flexible Modeling:** Our methods relax restrictions, providing flexibility in modeling various problems. Reducing primary conditions allows us to describe practical problems more freely, particularly when dealing with large samples.
2) **Simplified Application:** Restructuring Abu-Gadairi's approaches using graph data directly, bypassing the need for configuring topologies, simplifies application to extensive datasets. This adaptation, as seen in the medical application involving 10 patients, streamlines utilization, especially for non-topology specialists.
3) **Enhanced Accuracy:** Compared to prior methods, our approaches expand approximations and accuracy measures, facilitating more precise decisions in real-life scenarios like medical diagnosis (e.g., COVID-19 variants, ML applications, decision-making problems).
4) **High Accuracy:** Our proposed techniques, applied in diagnosing COVID-19 variants, outperformed previous works, yielding 100 % accuracy when compared to medical data in the decision table (Table 8).
5) **Algorithm Implementation:** An algorithm, accompanied by a flowchart and tested with simulated data, presents a straightforward method applicable in MATLAB, offering a comparative advantage over existing techniques.

### 7.2 Future Directions

1) **Further Applications:** Apply our approaches to additional medical and economic domains [26,35,39] to explore their efficacy in diverse real-world settings.
2) **Framework Expansion:** Emphasize the utilization of nearly j-initial approximations in various frameworks such as soft rough sets [27,28], rough fuzzy sets [29], fuzzy topological spaces [40], and graph theory applications [41].
3) **Exploring Integration:** Explore collaborative opportunities to integrate likelihood-based methods, like maximizing posterior or likelihood functions, with our proposed approach centered around nearly j-initial approximations. Anticipate enhanced precision and practical outcomes in real-life applications through this integration, marking it for inclusion in our upcoming research initiatives.

## Data availability

The used data in this study can be found in Ref. [38].

## CRediT authorship contribution statement

**Radwan Abu-Gdairi:** Writing – review & editing, Visualization, Validation, Funding acquisition, Data curation. **Mostafa K. El-Bably:** Writing – review & editing, Writing – original draft, Supervision, Formal analysis, Data curation, Conceptualization.

## Declaration of competing interest

The authors declare that they have no known competing financial interests or personal relationships that could have appeared to influence the work reported in this paper.
